# Limb Ischemic Postconditioning Alleviates Postcardiac Arrest Syndrome through the Inhibition of Mitochondrial Permeability Transition Pore Opening in a Porcine Model

**DOI:** 10.1155/2020/9136097

**Published:** 2020-04-15

**Authors:** Zhengquan Wang, Lifeng Wu, Jiefeng Xu, Jindan Gao, Sen Ye, Zilong Li, Yuanzhuo Chen, Xiangyu Zhang

**Affiliations:** ^1^Department of Critical Care Medicine, Shanghai Tenth People's Hospital, Tongji University School of Medicine, Shanghai, China; ^2^Department of Emergency Medicine, Yuyao People's Hospital, Medical School of Ningbo University, Ningbo, China; ^3^Department of Emergency Medicine, Second Affiliated Hospital, Zhejiang University School of Medicine, Hangzhou, China

## Abstract

**Objective:**

Previously, the opening of mitochondrial permeability transition pore (mPTP) was confirmed to play a key role in the pathophysiology of postcardiac arrest syndrome (PCAS). Recently, we demonstrated that limb ischemic postconditioning (LIpostC) alleviated cardiac and cerebral injuries after cardiac arrest and resuscitation. In this study, we investigated whether LIpostC would alleviate the severity of PCAS through inhibiting mPTP opening.

**Methods:**

Twenty-four male domestic pigs weighing 37 ± 2 kg were randomly divided into three groups: control, LIpostC, and LIpostC+atractyloside (Atr, the mPTP opener). Atr (10 mg/kg) was intravenously injected 30 mins prior to the induction of cardiac arrest. The animals were subjected to 10 mins of untreated ventricular fibrillation and 5 mins of cardiopulmonary resuscitation. Coincident with the beginning of cardiopulmonary resuscitation, LIpostC was induced by four cycles of 5 mins of limb ischemia and then 5 mins of reperfusion. The resuscitated animals were monitored for 4 hrs and observed for an additional 68 hrs.

**Results:**

After resuscitation, systemic inflammation and multiple organ injuries were observed in all resuscitated animals. However, postresuscitation systemic inflammation was significantly milder in the LIpostC group than in the control group. Myocardial, lung, and brain injuries after resuscitation were significantly improved in the LIpostC group compared to the control group. Nevertheless, pretreatment with Atr abolished all the protective effects induced by LIpostC.

**Conclusion:**

LIpostC significantly alleviated the severity of PCAS, in which the protective mechanism was associated with the inhibition of mPTP opening.

## 1. Introduction

Cardiac arrest (CA) remains one of the leading causes of death worldwide. It is estimated that 356,500 individuals in America and 550,000 individuals in China experience out-of-hospital CA every year, and the rates of survival to hospital discharge are 11.4% and less than 1%, respectively [1, 2]. Following global ischemia reperfusion (IR) triggered by CA and return of spontaneous circulation (ROSC), the ensuing postcardiac arrest syndrome (PCAS) would lead to brain damage, myocardial dysfunction, systemic inflammatory response, and finally multiple organ failure and death [3, 4]. Currently, one of the main contents of the post-CA care is the assessment and mitigation of IR injury to multiple organ systems [5].

Mitochondrial permeability transition pore (mPTP), regarded as a key factor in cell death following IR, has been confirmed to play an important role in the pathophysiology of PCAS [6, 7]. An early study demonstrated that the susceptibility of mPTP opening was increased accompanied with the impairment of mitochondrial respiration in multiple vital organs after resuscitation, and the inhibition of mPTP opening improved mitochondrial dysfunction and multiple organ failure [7]. A recent study further demonstrated that therapeutic hypothermia prevented postresuscitation mitochondrial dysfunction and attenuated the severity of PCAS by inhibiting mPTP opening [8]. Thus, mPTP might become a feasible target for therapeutic interventions to postresuscitation multiple organ dysfunction.

Limb ischemic postconditioning (LIpostC), which emerged as an endogenous form of organ protection, has been proven to provide potent myocardial and cerebral protection after CA and resuscitation. Earlier, one investigation demonstrated that LIpostC attenuated postresuscitation myocardial and cerebral dysfunctions and improved the duration of survival in rats [9]. Subsequently, another two investigations demonstrated that LIpostC reduced serum concentrations of biomarkers of cardiac and brain damage and facilitated their functional recovery after resuscitation in pigs [10, 11]. However, it is unknown whether LIpostC can inhibit mPTP opening so as to alleviate the severity of PCAS.

In this study, we investigated the protective effects of LIpostC on PCAS and its potential mechanism in a porcine model of CA and resuscitation. We hypothesized that LIpostC would alleviate systemic inflammation and myocardial, lung, and brain injuries after resuscitation, and these protective effects of LIpostC would be mediated by the inhibition of mPTP opening.

## 2. Materials and Methods

### 2.1. Study Design

This was a prospective, randomized, and controlled experimental study. A porcine model of CA and resuscitation was utilized. All the experimental procedures were performed based on the methods of our previous two studies, in which the animal model has been well established [10, 12]. The study was conducted in accordance with the requirements indicated in the guidelines of Institutional Animal Care and Use Committees, after the approval from the Ethics Committee of the Medical School of Ningbo University (approval number: IACUC-P201706).

### 2.2. Animal Preparation

All pigs were fasted overnight but allowed to drink water. Anesthesia was initiated by intramuscular injection of ketamine (20 mg/kg) and then an ear vein injection of sodium pentobarbital (30 mg/kg). After that, sodium pentobarbital (8 mg/kg/h) was continuously infused to maintain anesthesia. The endotracheal tube was advanced into the trachea. The animals were ventilated with a volume-controlled ventilator (SynoVent E5, Mindray, Shenzhen, China) with a tidal volume of 15 ml/kg, peak flow of 40 l/min, and FiO_2_ of 0.21. End-tidal carbon dioxide (ETCO_2_) was continuously monitored with a handheld ETCO_2_/SPO_2_ monitor (PMSH-300, SunLife Science Inc., Shanghai, China), and the breath rate was adjusted to maintain ETCO_2_ between 35 and 40 mmHg. The conventional lead II electrocardiogram was continuously monitored.

For the measurements of stroke volume (SV), global ejection fraction (GEF), extravascular lung water index (ELWI), and pulmonary vascular permeability index (PVPI), the PiCCO Monitor system (PiCCOplus, Pulsion Medical Systems, Munich, Germany) was used. First, a 7 Fr central venous catheter was inserted into the right internal jugular vein for the injection of iced saline. Second, a 4 Fr thermistor-tipped arterial catheter was inserted into the left femoral artery. Both of them were connected to the PiCCO Monitor system. For the measurement of aortic pressure and the collection of blood samples, a fluid-filled 8 Fr catheter (Model 6523, C.R. Bard Inc., Salt Lake, UT) was inserted from the right femoral artery into the thoracic aorta. For the measurement of right atrial pressure and the collection of blood samples, another fluid-filled 7 Fr catheter was inserted from the right femoral vein into the right atrium. All catheters were intermittently flushed with saline containing 5 IU bovine heparin per ml. For inducing ventricular fibrillation (VF), a 5 Fr pacing catheter (EP Technologies Inc., Mountain View, CA) was inserted from the right external jugular vein into the right ventricle. The position of catheters was confirmed by characteristic pressure morphology and with fluoroscopy. For the measurement of body temperature, a thermal probe was inserted into the rectum. Body temperature was maintained at 37.5 ± 0.5°C with the aid of the Blanketrol III Hyper-Hypothermia System (Cincinnati Sub-Zero, Cincinnati, OH).

### 2.3. Experimental Procedure

#### 2.3.1. Animal Randomization

After the surgical preparation was completed, the animals were stabilized for 15 mins. Baseline measurements were then acquired. The animals were randomized with the sealed envelope method into one of the three groups: control, LIpostC, and LIpostC+atractyloside (Atr).

#### 2.3.2. Induction of VF and CPR

VF was induced by 1 mA alternating current delivered to the right ventricular endocardium, and then, mechanical ventilation was discontinued. After 10 mins of untreated VF, CPR was manually performed by a ratio of 30 : 2 of compression to ventilation. The compression quality was monitored by an E Series Monitor Defibrillator (ZOLL Medical Corporation, Chelmsford, MA) to guarantee a compression depth of 50-60 mm at a rate of 100-120 per minute. The ventilation was performed by a bag respirator connected to the endotracheal tube. After 2.5 mins of CPR, a dose of 20 *μ*g/kg of epinephrine was intravenously administered. After 5 mins of CPR, the defibrillation was delivered with a single 150 J biphasic waveform electrical shock. If an organized rhythm with a mean arterial pressure (MAP) of greater than 50 mmHg was achieved for 5 mins or more, the animal was regarded as ROSC. With failure to achieve ROSC, manual CPR was immediately resumed for 2 mins followed by another defibrillation. This protocol was repeated until successful resuscitation or for a maximum of 10 mins. The same dose of epinephrine was administered every 3 mins after the first injection.

#### 2.3.3. Application of LIpostC and Atr

Thirty minutes prior to inducing VF, a dose of 10 mg/kg of Atr used as the mPTP opener was intravenously injected. Coincident with the beginning of cardiopulmonary resuscitation (CPR), LIpostC was induced by four cycles of 5 mins of limb ischemia and then 5 mins of reperfusion. Limb ischemia was induced by inflating a 5 cm wide blood pressure cuff around the upper third of the bilateral upper limb to stop the arterial blood supply. During the ischemic period, the cuff was inflated to 200 mmHg for 5 mins; the reperfusion was implemented by deflation for 5 mins.

#### 2.3.4. Postresuscitation Care

Following ROSC, mechanical ventilation was continued for 4 hrs. After that, the catheters were removed and the wounds were surgically sutured. When the animal recovered from anesthesia and had spontaneous respiration, the endotracheal tube was removed. The animals were then returned to their cages for a period of 68 hrs of observation, in which they had free access to water and food but no other treatments. At 72 hrs after resuscitation, the animals were euthanized with an intravenous injection of sodium pentobarbital (150 mg/kg). A routine necropsy was performed for documentation of possible injuries to the bony thorax and thoracic and abdominal viscera resulting from the surgical or CPR intervention or the presence of obfuscating diseases.

### 2.4. Measurement

Aortic and right atrial pressures, electrocardiogram, and pulse oxygen saturation were continuously measured by a patient monitoring system (BeneView T6, Mindray, Shenzhen, China). Coronary perfusion pressure (CPP) was calculated as the difference between decompression diastolic aortic and time-coincident right atrial pressures. The outcomes of CPR including ROSC, duration of CPR, number of defibrillations, and epinephrine dosage were recorded.

Artery blood gases and lactate were measured at the baseline and at 2 hrs and 4 hrs after resuscitation on 1.5 ml of arterial blood samples with a Blood Gas/Electrolyte Analyzer (Model 5700, Instrumentation Laboratory, Lexington, MA). PaO_2_/FiO_2_ was calculated as the ratio of PaO_2_ and FiO_2_. SV and GEF, as the indexes of myocardial function, were measured at the baseline and hourly after resuscitation for a total of 4 hrs with the PiCCO Monitor system. ELWI and PVPI, as the indexes of lung water, were measured at the same time points with the PiCCO Monitor system. Venous blood samples were collected at the baseline and at 2 hrs, 4 hrs, 24 hrs, 48 hrs, and 72 hrs after resuscitation, and then, the serums were separated and stored at -80°C until analysis. The serum levels of tumor necrosis factor-*α* (TNF-*α*), interleukin-6 (IL-6), and cardiac troponin I (cTnI) at the baseline and at 2 hrs, 4 hrs, and 24 hrs after resuscitation were measured with enzyme-linked immunosorbent assay kits (Meixuan Biotechnology Inc., Shanghai, China) according to the manufacturer's instructions. The serum levels of neuron-specific enolase (NSE) and S100B protein (S100B) at the baseline and at 24 hrs, 48 hrs, and 72 hrs after resuscitation were measured with the same method. Postresuscitation neurologic function was evaluated according to the method of neurological deficit score (NDS) at 24 hr intervals for a total of 72 hrs. The NDS was scored from 0 (no observed neurologic deficit) to 400 (death or brain death) [13], which was examined and confirmed by two investigators who were blinded to the study.

For the measurements of inflammatory and oxidative injuries in the heart, lung, and brain, the left ventricular myocardium, right lower lung lobe, and cerebral cortex were harvested at 72 hrs postresuscitation. All tissues were washed in 4°C of cold saline, then frozen in liquid nitrogen, and finally stored at -80°C for further analysis. All specimens were homogenized with normal saline on ice and centrifuged at 4000 rpm at 4°C for 15 mins. The supernatants were collected for measuring the levels of TNF-*α*, IL-6, and malondialdehyde (MDA) and the activities of superoxide dismutase (SOD). The inflammatory cytokines were measured with enzyme-linked immunosorbent assay kits (Meixuan Biotechnology Inc., Shanghai, China). The contents of MDA were determined by the thiobarbituric acid reactive substance assay, and the activities of SOD were determined by the xanthine oxide assay [14]. All the assay kits were purchased from Nanjing Jiancheng Bioengineering Institute (Nanjing, China). The results were expressed as per microgram of protein.

### 2.5. Statistical Analysis

The estimation of sample size was performed by power analysis, in which the number of at least six animals in each group was needed to get a power of more than 80% for all variables. Continuous variables were shown as the mean ± standard deviation for normally distributed data or as median (25th and 75th percentiles) for nonnormally distributed data. Normal distribution was confirmed with the Kolmogorov-Smirnov test. Variables were compared with one-way analysis of variance or the Kruskal-Wallis test for nonparametric data. Comparisons between time-based measurements within each group were performed with repeated measures analysis of variance. If there was a significant difference in the overall comparison of groups, comparisons between any other two groups were made by the Bonferroni test. For the comparison of categorical variables such as ROSC, Fisher's exact test was used. A value of *P* < 0.05 was considered significant.

## 3. Results

**Figure 1:**
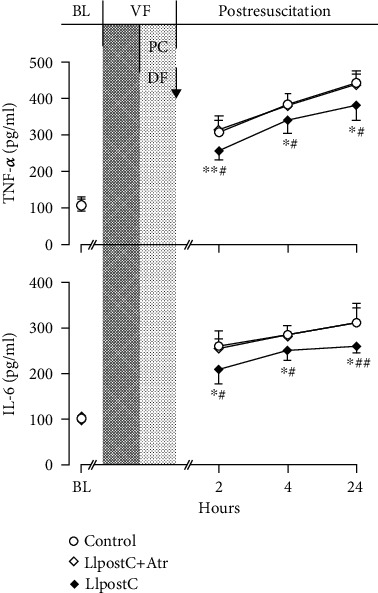
The changes of serum tumor necrosis factor-*α* (TNF-*α*) and interleukin-6 (IL-6) in the different groups. BL: baseline; VF: ventricular fibrillation; PC: precordial compression; DF: defibrillation; LIpostC: limb ischemic postconditioning; Atr: atractyloside. ^∗^*P* < 0.05 and ^∗∗^*P* < 0.01 versus the control group; ^#^*P* < 0.05 and ^##^*P* < 0.01 versus the LIpostC+Atr group.

**Figure 2:**
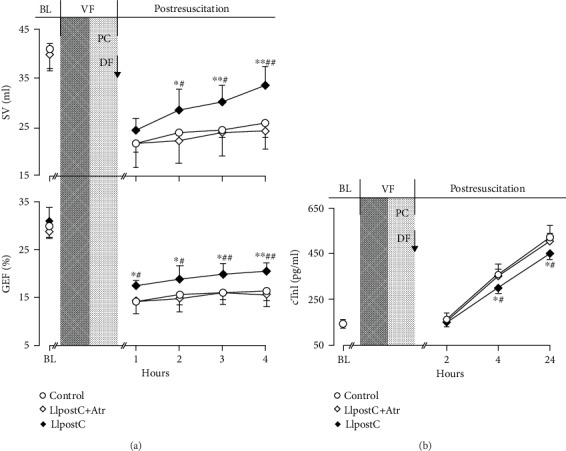
The changes of stroke volume (SV), global ejection fraction (GEF), and serum cardiac troponin I (cTnI) in the different groups: (a) SV and GEF; (b) cTnI. BL: baseline; VF: ventricular fibrillation; PC: precordial compression; DF: defibrillation; LIpostC: limb ischemic postconditioning; Atr: atractyloside. ^∗^*P* < 0.05 and ^∗∗^*P* < 0.01 versus the control group; ^#^*P* < 0.05 and ^##^*P* < 0.01 versus the LIpostC+Atr group.

**Figure 3:**
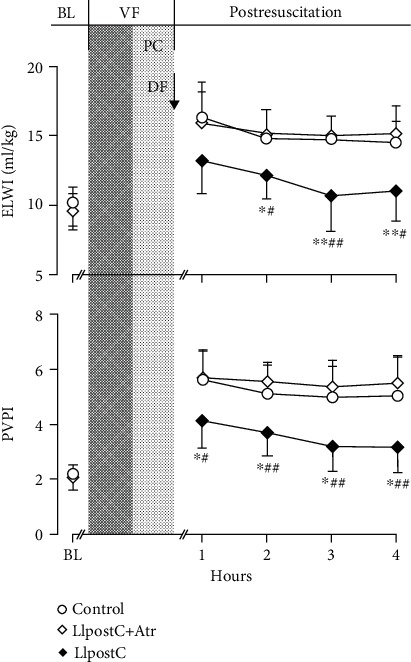
The changes of extravascular lung water index (ELWI) and pulmonary vascular permeability index (PVPI) in the different groups. BL: baseline; VF: ventricular fibrillation; PC: precordial compression; DF: defibrillation; LIpostC: limb ischemic postconditioning; Atr: atractyloside. ^∗^*P* < 0.05 and ^∗∗^*P* < 0.01 versus the control group; ^#^*P* < 0.05 and ^##^*P* < 0.01 versus the LIpostC+Atr group.

**Figure 4:**
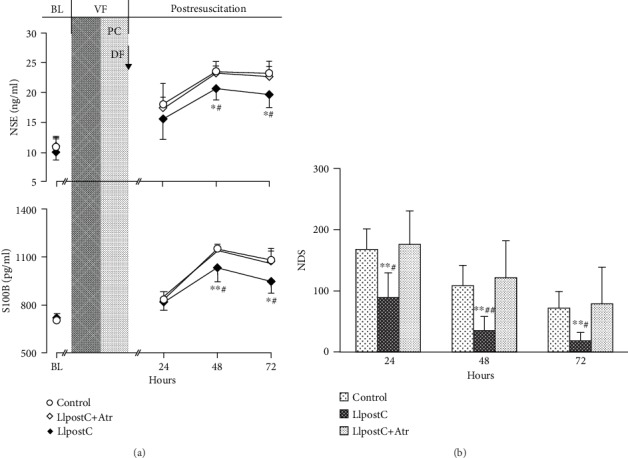
The changes of serum neuron-specific enolase (NSE) and S100B protein (S100B) and neurological deficit score (NDS) in the different groups: (a) NSE and S100B; (b) NDS. BL: baseline; VF: ventricular fibrillation; PC: precordial compression; DF: defibrillation; LIpostC: limb ischemic postconditioning; Atr: atractyloside. ^∗^*P* < 0.05 and ^∗∗^*P* < 0.01 versus the control group; ^#^*P* < 0.05 and ^##^*P* < 0.01 versus the LIpostC+Atr group.

In total, twenty-four studies were performed and completed. There were no significant differences on baseline hemodynamics, myocardial function, lung water, and blood analytical measurements among the three groups (Tables 1 and 2, Figures 1–4).

During CPR, the similar levels of CPP were achieved in all animals experiencing CA and resuscitation. Consequently, seven of the eight animals in the LIpostC and control groups and six of the eight animals in the LIpostC+Atr group were successfully resuscitated, in which the rate of ROSC was not significant among the three groups. In addition, no significant differences were observed in duration of CPR, number of defibrillations, and dosage of epinephrine among the three groups (Table 3). Finally, all the resuscitated animals survived for 72 hrs in the three groups.

After resuscitation, pH was decreased and lactate was increased in all the resuscitated animals. However, the values of pH were greater in the LIpostC group than in the other two groups, in which the difference was significant at 2 hrs postresuscitation between the LIpostC and control groups. In addition, the values of lactate were significantly lower at 2 and 4 hrs postresuscitation in the LIpostC group compared with the other two groups. Postresuscitation PO_2_ was also decreased in all the resuscitated animals. However, the levels of PO_2_ were gradually increased after resuscitation in the LIpostC group, which were significantly greater at 2 hrs and 4 hrs compared to the other two groups. There were no significant differences in arterial PCO_2_ and HCO_3_^−^ among the three groups (Table 2).

After resuscitation, the levels of TNF-*α* and IL-6 in serum were significantly increased in all the resuscitated animals when compared to the baseline levels. However, the increases in TNF-*α* and IL-6 were significantly milder starting 2 hrs after resuscitation in the LIpostC group when compared with the other two groups Figure 1.

After resuscitation, SV and GEF were decreased, and meanwhile, cTnI were increased in all the resuscitated animals. However, a faster improvement in them was observed in the LIpostC group than in the other two groups. Especially in the LIpostC group, the values of SV and GEF were significantly greater starting 2 hrs after resuscitation and the serum levels of cTnI were significantly lower starting 4 hrs after resuscitation when compared to the other two groups Figure 2.

After resuscitation, ELWI and PVPI were significantly increased, and meanwhile, PaO_2_/FiO_2_ was significantly decreased in all the resuscitated animals when compared to the baseline levels. However, the values of ELWI and PVPI were significantly decreased while the values of PaO_2_/FiO_2_ were significantly increased starting 2 hrs after resuscitation in the LIpostC group compared to the other two groups (Figure 3).

After resuscitation, NSE and S100B were significantly increased in all the resuscitated animals when compared to the baseline levels. However, significantly lower levels of NSE and S100B in serum were observed at 48 hrs and 72 hrs after resuscitation in the LIpostC group than in the other two groups. In addition, NDS was significantly better at 24 hrs, 48 hrs, and 72 hrs after resuscitation in the LIpostC group (Figure 4).

**Figure 5:**
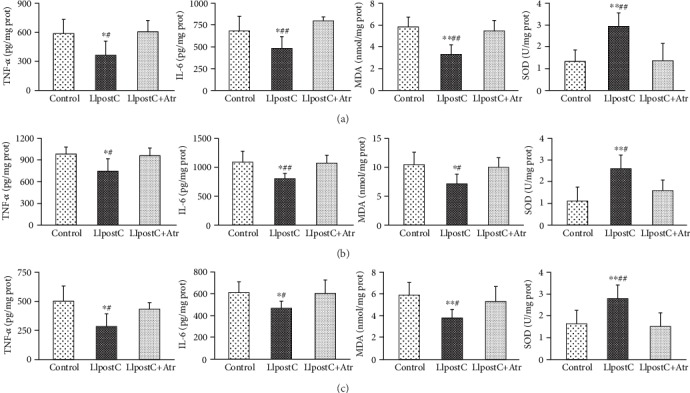
The comparisons of tissue inflammation and oxidative stress in the heart, lung, and brain in the different groups. (a) The levels of tumor necrosis factor-*α* (TNF-*α*), interleukin-6 (IL-6), and malondialdehyde (MDA) and the activity of superoxide dismutase (SOD) in the heart. (b) The levels of TNF-*α*, IL-6, and MDA and the activity of SOD in the lung. (c) The levels of TNF-*α*, IL-6, and MDA and the activity of SOD in the brain. LIpostC: limb ischemic postconditioning; Atr: atractyloside. ^∗^*P* < 0.05 and ^∗∗^*P* < 0.01 versus the control group; ^#^*P* < 0.05 and ^##^*P* < 0.01 versus the LIpostC+Atr group.

At 72 hrs postresuscitation, no significant abnormalities were observed on gross examination at necropsy in all the resuscitated animals. However, the levels of TNF-*α* and IL-6 in the heart, lung, and brain were significantly lower in the LIpostC group than in the other two groups. In addition, the levels of MDA were significantly lower while the activities of SOD were significantly greater in the heart, lung, and brain in the LIpostC group compared to the other two groups (Figure 5).

## 4. Discussion

The present study demonstrated that LIpostC significantly alleviated postresuscitation systemic inflammation and myocardial, lung, and brain injuries, in which the alleviation of multiple organ injuries was related to the inhibition of tissue inflammation and oxidative stress. However, the pretreatment of the mPTP opener completely abolished the protective effects of LIpostC on PCAS. These results indicated that LIpost alleviated the severity of PCAS through the inhibition of mPTP opening.

After restoring spontaneous circulation, most immediate survivors would die of PCAS [4]. The poor outcomes of PCAS are mainly attributed to postresuscitation systemic inflammation and multiple organ dysfunctions, in which cerebral, cardiovascular, and respiratory impairments contribute to the high in-hospital mortality in CA victims [4, 15]. New therapeutic interventions are urgently needed to effectively mitigate the damage of these organ systems so as to improve the prognosis of patients. Recently, a series of studies demonstrated that LIpostC was a feasible approach to produce active organ protection and significantly improved cardiac and neurological outcomes after CA and resuscitation [9–11]. In the present study, we applied the same algorithms of LIpostC to explore its protective effects on PCAS. Consequently, postresuscitation systemic inflammation and myocardial, lung, and brain injuries were observed in all the survived animals. However, LIpostC significantly alleviated systemic inflammation and multiple organ injuries when compared with the control group. In addition, pathologic analysis indicated that tissue inflammation and oxidative stress in the heart, lung, and brain after resuscitation were significantly milder in the LIpostC group than in the control group. Thus, LIpostC may become a promising method for postresuscitation multiple organ protection in the clinical setting.

mPTP is one kind of nonselective pores formed by multiprotein complexes in the inner membrane of the mitochondria. When the mPTP is opened, the electrochemical gradient across the inner membrane would be disrupted, which then results in matrix swelling and rupture of the outer membrane of mitochondria and finally the release of proapoptotic substances from mitochondria [16]. The released cytochrome c would induce the cascade of caspase activation and finally cause cell death [17, 18]. Currently, a series of studies have manifested that mPTP participates in the pathophysiology of PCAS and might be a therapeutic target for postresuscitation multiple organ dysfunction. In a pig model of CA, Gong et al. [19] demonstrated that therapeutic hypothermia provided postresuscitation neurological protection by reducing the mPTP opening and its related cell apoptosis. In a rat model of CA, Knapp et al. [20] demonstrated that pharmacological postconditioning with the mPTP inhibitor attenuated postresuscitation myocardial dysfunction and neurological damage. In a rabbit model of CA, Cour et al. [7, 21] demonstrated that the inhibition of mPTP opening with cyclosporine A or NIM 811 prevented postresuscitation multiple organ failure and improved short-term survival. In another rabbit model of CA, Jahandiez et al. [8] demonstrated that therapeutic hypothermia was as effective as NIM 811 to limit the severity of PCAS by inhibiting the opening of mPTP.

Currently, no investigation has explored the effects of LIpostC on mPTP in a CA model. However, a series of studies have fully elucidated that mPTP is involved in organ protection of ischemic postconditioning in animal models of regional organ IR injury. Bochaton et al. [22] demonstrated that cardiac ischemic postconditioning prevented lethal IR injury by inhibiting cyclophilin D-mediated mPTP opening. Lin et al. [23] demonstrated that ischemic postconditioning attenuated cell deaths after liver IR injury, and the protective effects were weakened by another mPTP opener—Atr. Sun et al. [24] demonstrated that ischemic postconditioning reduced infarction volume and improved the neurological outcome after cerebral IR injury, while the use of Atr reversed its neuroprotective effects. Similarly, Cheng et al. [25] demonstrated that the protective effects of ischemic postconditioning after intestinal IR injury were negated by the addition of Atr. In an attempt to confirm the potential mechanism of organ protection produced by LIpostC after resuscitation, the Atr was chosen to open the mPTP in the present study. A dose of 10 mg/kg of Atr was used based on a previous study [26], in which Atr abolished the protective effects of ischemic postconditioning on ischemic skeletal muscle in a pig model. Consequently, significant decreases in postresuscitation systemic inflammation and multiple organ injuries produced by LIpostC did not occur when pretreating with the mPTP opener in the LIpostC+Atr group. These results indicated that the inhibition of mPTP opening was involved in multiple organ protection of LIpostC after CA and resuscitation.

There were several potential limitations in our study. First, although remote ischemic conditioning was successfully implemented in this porcine model, its optimal algorithms are still unknown and require further investigations. Second, we observed that LIpostC produced a short-term protection against PCAS during the 72 hrs of postresuscitation observation. The evaluation of long-term protection of LIpostC is needed in the future animal studies and clinical trials. Third, we demonstrated that the inhibition of mPTP opening was involved in the protective mechanism of LIpostC; however, its downstream signalling pathways were not investigated in this study.

## 5. Conclusion

LIpostC alleviated the severity of PCAS through the inhibition of the mPTP opening in a porcine model of CA and resuscitation.

## Figures and Tables

**Table 1:** Baseline characteristics.

Group	Control (*n* = 8)	LIpostC (*n* = 8)	LIpostC+Atr (*n* = 8)
Body weight (kg)	37.6 ± 2.1	36.6 ± 1.3	37.3 ± 2.9
Heart rate (beats/min)	115 ± 10	110 ± 5	114 ± 5
Mean arterial pressure (mmHg)	113 ± 6	109 ± 7	109 ± 9
End-tidal CO_2_ (mmHg)	40.8 ± 1.3	40.6 ± 1.5	39.9 ± 2.1
Body temperature (°C)	37.4 ± 0.3	37.4 ± 0.5	37.5 ± 0.3

LIpostC: limb ischemic postconditioning; Atr: atractyloside. Values are presented as the mean ± standard deviation.

**Table 2:** Arterial blood gas and lactate.

	Baseline	Postresuscitation	
		2 h	4 h
pH			
Control	7.49 ± 0.05	7.32 ± 0.09	7.41 ± 0.08
LIpostC	7.48 ± 0.06	7.43 ± 0.06^∗^	7.47 ± 0.05
LIpostC+Atr	7.49 ± 0.04	7.38 ± 0.06	7.45 ± 0.05
PO_2_ (mmHg)			
Control	93 ± 12	58 ± 11	55 ± 12
LIpostC	93 ± 10	74 ± 7^∗^^,#^	81±5^∗∗^^,#^
LIpostC+Atr	88 ± 12	60 ± 9	62 ± 17
PCO_2_ (mmHg)			
Control	39 ± 5	50 ± 10	47 ± 10
LIpostC	40 ± 5	45 ± 8	43 ± 7
LIpostC+Atr	41 ± 7	48 ± 6	42 ± 3
HCO_3_^−^ (mmol/l)			
Control	29 ± 2	26 ± 6	29 ± 4
LIpostC	31 ± 5	30 ± 3	31 ± 4
LIpostC+Atr	32 ± 3	28 ± 5	30 ± 3
PaO_2_/FiO_2_ (mmHg)			
Control	443 ± 59	274 ± 51	263 ± 59
LIpostC	444 ± 48	351 ± 38^∗^^,#^	388±24^∗∗^^,#^
LIpostC+Atr	420 ± 59	286 ± 41	293 ± 80
Lactate (mmol/l)			
Control	1.3 ± 0.4	5.0 ± 2.5	2.7 ± 1.2
LIpostC	1.3 ± 0.3	2.2 ± 1.2^∗^^,#^	1.2 ± 0.7^∗^^,#^
LIpostC+Atr	1.1 ± 0.3	4.6 ± 1.6	2.5 ± 1.2

LIpostC: limb ischemic postconditioning; Atr: atractyloside. Values are presented as the mean ± standard deviation. ^∗^*P* < 0.05 and ^∗∗^*P* < 0.01 versus the control group; ^#^*P* < 0.05 versus the LIpostC+Atr group.

**Table 3:** Cardiopulmonary resuscitation outcomes.

Group	Control	LIpostC	LIpostC+Atr
CPP in PC1 (mmHg)	20.3 ± 2.9	20.7 ± 3.0	19.9 ± 2.9
CPP in PC3 (mmHg)	30.6 ± 5.9	28.9 ± 4.0	29.4 ± 4.0
CPP in PC5 (mmHg)	27.0 ± 7.1	27.9 ± 4.9	26.4 ± 5.0
ROSC (*n*/*n*)	7/8	7/8	6/8
Duration of CPR (min)	5 (5, 7)	5 (5, 7)	7 (5, 15)
Number of defibrillation (*n*)	1 (1, 2)	1 (1, 2)	2 (1, 6)
Epinephrine dosage (mg)	0.80 (0.74, 1.52)	0.74 (0.72, 1.48)	1.40 (0.76, 3.60)

LIpostC: limb ischemic postconditioning; Atr: atractyloside; CPP: coronary perfusion pressure; PC*n*: *n* min after precordial compression; ROSC: return of spontaneous circulation; CPR: cardiopulmonary resuscitation. Values are presented as the mean ± standard deviation or median (25th and 75th percentiles).

## Data Availability

All the data are available and will be submitted if required.
